# Enhanced BBB penetration and microglia-targeting nanomodulator for the two-pronged modulation of chronically activated microglia-mediated neuroinflammation in Alzheimer's disease

**DOI:** 10.1016/j.apsb.2025.01.015

**Published:** 2025-01-25

**Authors:** Ya Wei, Xue Xia, Xiaorong Wang, Wenqin Yang, Siqin He, Lulu Wang, Yongke Chen, Yang Zhou, Feng Chen, Hanmei Li, Fu Peng, Guobo Li, Zheng Xu, Jintao Fu, Huile Gao

**Affiliations:** a Key Laboratory of Tropical Biological Resources of Ministry of Education, School of Pharmaceutical Sciences, Hainan University, Haikou 570200, China; b Key Laboratory of Drug-Targeting and Drug Delivery System of the Education Ministry, West China School of Pharmacy, Sichuan University, Chengdu 610041, China; c Department of Radiology, Hainan General Hospital (Hainan Affiliated Hospital of Hainan Medical University), Haikou 570311, China; d School of Food and Biological Engineering, Chengdu University, Chengdu 610106, China; e State Key Laboratory of Biotherapy, West China Hospital, Sichuan University, Chengdu 610041, China

**Keywords:** Blood‒brain barrier penetration, Microglial targeting, Microglial modulation, Chronic neuroinflammation, Alzheimer's disease, Immunotherapy, TREM2, Resveratrol

## Abstract

Intervention in chronically activated microglia-mediated neuroinflammation is a novel approach to treat Alzheimer's disease (AD). The low permeability of the blood‒brain barrier (BBB) and non-selective distribution in the brain severely restrict AD drugs' disease-modifying efficacy. Here, an immunosuppressant TREM2-lowing antisense oligonucleotides (ASOs) and resveratrol co-loaded cationic liposome is developed as an immune reprogramming nanomodulator modified by acid-cleavable BBB-targeting peptide and microglia-targeting peptide (Res@TcMNP/ASO) for AD management. Res@TcMNP/ASO can enter brain endothelial cells *via* D-T7 peptides. Then D-T7 undergoes an acid-responsive cleavage, facilitating the escape of Res@MNP/ASO from endo/lysosomes to cross the BBB. The detached Res@MNP/ASO specifically targets M1-phenotype microglia *via* exposed MG1 peptides to prompt the simultaneous delivery of two drugs into activated microglia. This nanomodulator can not only restore the immune function of microglia through TREM2-lowing ASO but also mitigate the immune stimulation to microglia caused by reactive oxygen species (ROS) through resveratrol, thereby synergistically inhibiting the chronic activation of microglia to alleviate neuroinflammation in AD. Our results indicate that this combination treatment can achieve significant behavioral and cognitive improvements in late APP/PS1 mice.

## Graphical abstract

The nanomodulator can efficiently cross the BBB and co-deliver immunosuppressant TREM2-lowing antisense oligonucleotides and resveratrol towards M1 phenotype microglia, finally synergistically inhibiting chronically activated microglia for AD management.

**Figure:**
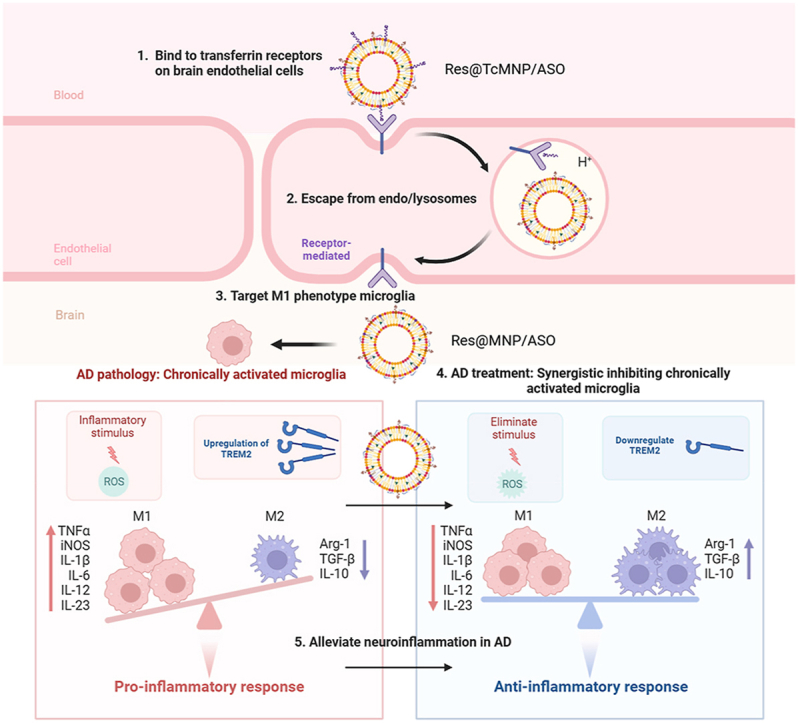

## 1 Introduction

Alzheimer's disease (AD) is a neurodegenerative disorder characterized by cognitive impairments, putting tremendous economic strain on both families and society^1^, ^2^, ^3^. Recent studies have identified chronically activated microglia-mediated neuroinflammation as one of the key pathogenic factors in AD^4^, ^5^, ^6^, ^7^, ^8^, ^9^. Specifically speaking, microglia, the innate immune cells in the brain, can activate into the neurotoxic M1 phenotype and the neuroprotective M2 phenotype^10^. At AD's initial phase, microglia are acutely activated to clear misfolded protein aggregates and cellular debris, which is generally protective. As AD progresses, microglia are chronically activated, leading to impaired phagocytosis and sustained release of proinflammatory mediators, establishing a detrimental crosstalk culminating in severe neuronal loss^11^, ^12^, ^13^, ^14^, ^15^, ^16^. Consequently, researchers are directing their attention toward regulating the chronic activation of microglia to inhibit pro-inflammatory immune responses in AD^17^^,^^18^.

Triggering receptor expressed on myeloid cells 2 (TREM2), a regulator of microglial immune function, is a type I transmembrane protein receptor^19^. It mediates the response of microglia to stimuli, thereby affecting the activation, phagocytosis, proliferation, and survival of microglia^20^, ^21^, ^22^. Chronically activated microglia are in a state of easily triggered and persistently activated dysfunction characterized by down-regulation of homeostatic genes and up-regulation of AD-related genes such as TREM2^11^^,^^12^^,^^14^^,^^18^. Inhibiting TREM2 expression in late APP/PS1 mice's brains can induce acute activation of microglia, thereby restoring the phagocytic capacity of microglia, limiting plaque deposition, and promoting an anti-inflammatory environment in the brain^23^. Various drugs have emerged for AD management against TREM2, including small molecule drugs^24^, antibody drugs^25^^,^^26^, and small nucleic acid drugs^23^. Small nucleic acid drugs are effective treatments for ameliorating transcriptional and translational changes associated with microglia because of their better targeting and specificity, longer-lasting effect, shorter development cycle, and less drug resistance^27^. Among them, antisense oligonucleotides (ASOs) are considered highly valuable therapeutic agents for interfering with down-regulated gene expression because of the advantages of reversibility, a wider mechanism of action, and the ability to act on both the nucleus and cytoplasm^28^. Thus, downregulating TREM2 by TREM2-lowing ASO could regulate dysfunctional microglia to inhibit the chronic activation of microglia. However, it has also been found that there is a close interaction between the overactivated immune microenvironment in the brain and microglia. Exogenous immune stimuli such as excessive reactive oxygen species (ROS) can also result in chronic activation of microglia, causing a pro-inflammatory immune response^7^^,^^9^^,^^29^. Thus, immune stimuli need to be eliminated to enhance the effect of regulating microglia. Owing to its distinctive polyphenolic hydroxyl structure, resveratrol could effectively scavenge exogenous immune stimuli ROS^30^^,^^31^. Summing up the above points, a combination of TREM2-lowing ASO and resveratrol can achieve a synergistic regulation of microglia phenotype by restoring the immune function of microglia and eliminating the immune stimulation of exogenous ROS, thereby providing effective neuroprotection against AD neuroinflammation.

Notably, for effective synergistic regulation of microglia, two drugs must simultaneously penetrate the blood‒brain barrier (BBB) and reach microglia. Receptor-mediated transcytosis combined with advancements in nanotechnology becomes a useful way to cross the BBB^32^, ^33^, ^34^, ^35^. However, nanoparticles (NPs) modified with high-affinity ligands can become trapped in endosomes/lysosomes, resulting in low efficiency across the BBB^36^, ^37^, ^38^. The acid-cleavable high-affinity D-T7, as demonstrated in our previous studies, exhibits the ability to dissociate from NPs within the acidic environment of endo/lysosomes, thereby significantly enhancing the efficiency of BBB penetration by NPs^39^, ^40^, ^41^, ^42^. In addition, the extensive brain distribution of drugs after crossing the BBB may cause side effects on the normal brain and reduce drugs’ therapeutic efficacy at the target site. Cascade-targeted NPs effectively overcome this widespread distribution defect and achieve high-precision targeted delivery in the brain^43^, ^44^, ^45^, ^46^. The MG1 peptide was chosen as the second targeting ligand due to its high affinity for the M1 phenotype microglia^47^ to meet the needs of targeting microglial.

**Scheme 1:**
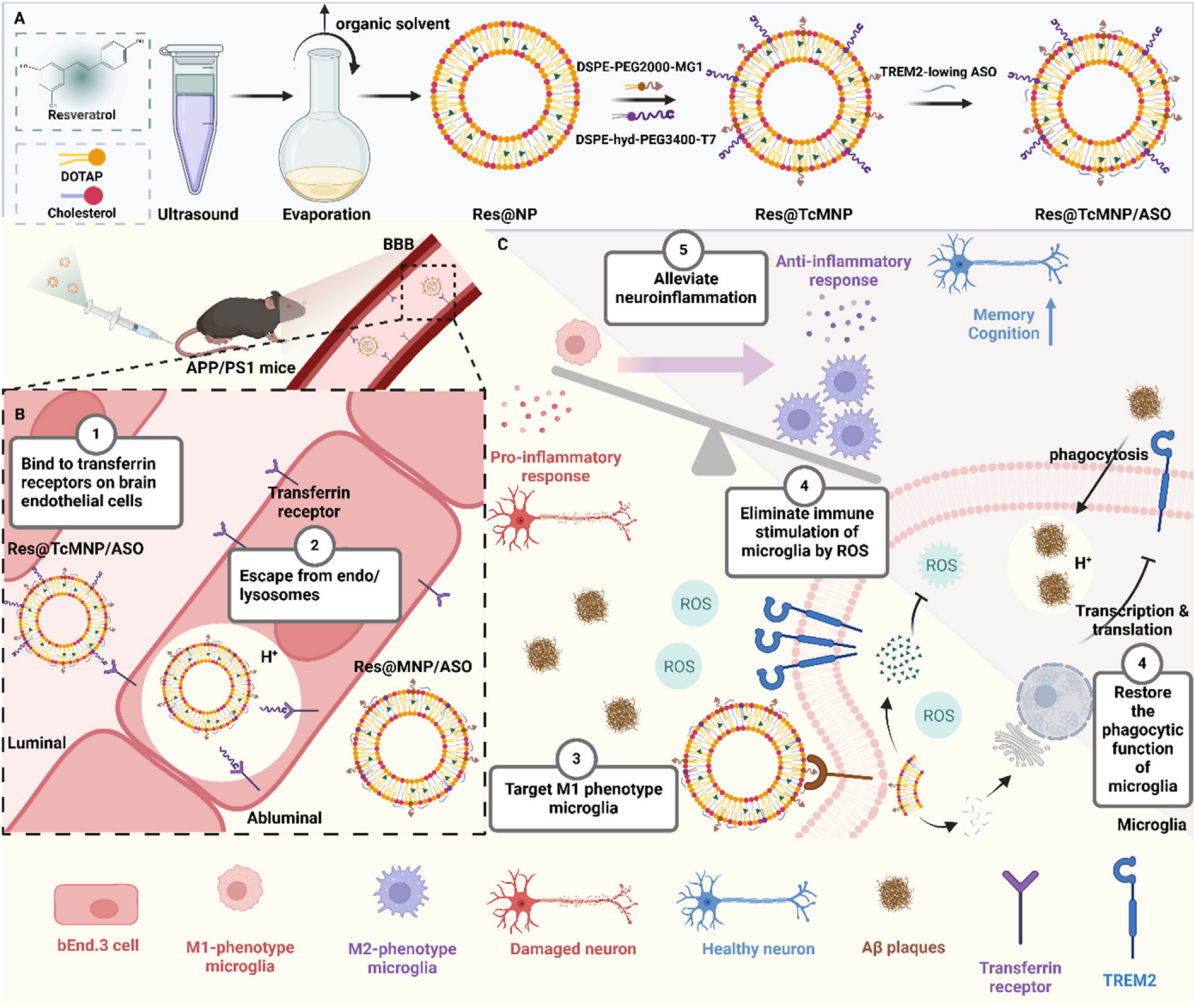
Schematic diagram of the strategy. (A) Diagram showing the formation of nanomodulator (Res@TcMNP/ASO). (B) Schematic illustration of Res@TcMNP/ASO penetrating the BBB. (C) Schematic illustration of the two-pronged modulation of chronically activated microglia-mediated neuroinflammation against AD by Res@TcMNP/ASO.

Based on the aforementioned information, we developed an acid-cleavable cascade targeting nanomodulator (Res@TcMNP/ASO) to co-delivery TREM2-lowing ASO and Res to M1 phenotype microglia. This nanomodulator is modified with D-T7 and MG1 on acid-cleavable long PEG chains and short PEG chains respectively (Scheme 1A). After intravenous administration, the high-affinity D-T7 peptides bind to transferrin receptors on brain endothelial cells. The long PEG chains are removed from Res@TcMNP/ASO in the acidic environment of endo/lysosomes, facilitating their conversion into Res@MNP/ASO and enhancing the brain penetration capability of Res@MNP/ASO (Scheme 1B). Finally, Res@MNP/ASO targeted M1 phenotype microglia *via* exposed MG1 peptides. The proposed approach facilitates the enhanced and simultaneous delivery of TREM2-lowing ASO and resveratrol to activated microglia, aiming to restore microglial immune function and eliminate immune stimulation. Consequently, this intervention synergistically alleviated neuroinflammation in AD (Scheme 1C).

## 2 Materials and methods

### 2.1 Materials

DSPE-PEG2000-MAL, DSPE-PEG3400-MAL, and DSPE-PEG2000-OME were bought from Ponsure Biotechnology (Shanghai, China). D-T7 peptides (sequence: HRPYIAHC, MW = 996.16, all D-form amino acids) and MG1 (sequence: CHHSSSAR, MW = 883.94) were derived from Shanghai Top-peptide Co., Ltd. (Shanghai, China). DSPE-hyd-PEG3400-MAL were derived from Xi'an Ruixi Biological Co., Ltd. (Xi'an, China). 2,3-Di (octadec-9-enoyloxy) propyl-trimethylammonium (DOTAP) and cholesterol came from AVT Pharmaceutical Tech Co., Ltd. (Shanghai, China). Resveratrol was obtained from TCI (Japan). The TREM2-lowing ASO (sense: ATGGAGTGTATCCCCGACGC) and negative control ASO (sense: CCTATAGGACTATCCAGGAA) were synthesized by Beijing Tsingke Biotech Co., Ltd. (Beijing, China). The TREM2 antibody came from Cell Signaling Technology (USA). A membrane protein extraction kit was bought from Beijing solarbio science&technology Co., Ltd. (Beijing, China). Fluoresbrite YG Microspheres (Polysciences) was derived from Beijing Biotyscience Technology Co., Ltd. (Beijing, China). Lipopolysaccharide (LPS) was obtained from Invitrogen (USA). IKB-*α* and Iba1 antibody was purchased from Abcam (UK). Enzyme-linked immunosorbent assay (ELISA) kits were bought from Thermo Fisher Scientific Inc (USA). *β*-Tubulin, SOD1, iNOS, Arg1, Nrf2, GCLM, NQO1, BDNF, and PSD95 antibodies came from ABclonal Technology Co., Ltd. (Wuhan, China). The bEnd.3, BV-2, SH-SY5Y cell lines came from the Chinese Academy of Sciences Cell Bank (Shanghai, China). B6-Tg (APPswePSEN1DE9) mice were derived from Beijing wesun Biotechnology Co., Ltd. (Beijing, China). All animal experiments were performed under the guidelines, evaluated, and approved by the ethics committee of Sichuan University (No. K2022011).

### 2.2 Synthesis, purification, and characterization of long-chain T7 peptide and short-chain MG1 peptide

DSPE-hyd-PEG3400-MAL was dissolved in DMF. D-T7 was dissolved in PBS with EDTA (0.1 mmol/L, pH = 8)*n* (D-T7): *n* (DSPE-hyd-PEG3400-Mal) = 1.5:1. Then, D-T7 solution and DSPE-hyd-PEG3400-Mal were mixed and stirred constantly at 25 °C under argon's protection away from light. Final products were dialyzed (MWCO 3500 Da) for 24 h in PBS (pH = 8). The dialysis media should be changed at least 4 times during this period. Next, a Millipore (MWCO 3000 Da) was used to wash the product 3 times with ultrapure water. Finally, the solution was lyophilized to obtain a white solid powder. For comparison, DSPE-PEG3400-T7 was synthesized. To mimic the entry of NPs into the acidic environment of lysosomes or endosomes, acid-cleavable fragments DSPE-hyd-PEG3400-T7 and acid-stable fragments DSPE-PEG3400-T7 were incubated with PBS at pH 5.5 at 37 °C overnight. The products were ultrafiltrated with an ultrafiltration tube (MWCO 3500 Da) and washed three times. Changes in molecular weight before and after were identified by matrix-assisted laser desorption/ionization-time of flight mass spectrum (MALDI-TOF-MS) (Bruker Daltonics, USA). The same reactant ratio and reaction conditions were used to synthesize short chains. The final product was dialyzed (MWCO 2000 Da) for 24 h in ultrapure water.

### 2.3 Preparation of Res@TcMNP/ASO and other control NPs

Res@TcMNP/ASO was fabricated using the emulsion/solvent evaporation method. In brief, a solution of lipids in CHCl_3_ (200 μL) containing cholesterol and DOTAP at a molar ratio of 1:1 was mixed with a solution of resveratrol (50 μL) in CH_3_OH (resveratrol/cholesterol and DOTAP = 1/15, *m*/*m*). Then the solution was mixed with ddH_2_O (1.9 mL) and emulsified by sonication at 100 W for 5 min in an ice water bath (5 s per ultrasound followed by 5 s pause). Thereafter, the solution which removed the organic solvent was incubated with ddH_2_O (0.1 mL, *n*(DSPE-PEG2000-MG1):(DSPE-hyd-PEG3400-T7):*n*(DSPE-PEG2000-OME) = 1:1:3) for 30 min at 37 °C. After ultrafiltration at 5500 rpm for 15 min at 4 °C and washing with ddH_2_O, TcMNP/ASO@Res was prepared by adding TREM2-lowing ASOs to the NPs solution. NP does not modify T7 peptides and MG1 peptides. TNP modifies acid-insensitive T7 peptides but not MG1 peptides. MNP modifies MG1 peptides but not T7 peptides. TcNP modifies acid-sensitive T7 peptides but not MG1 peptides. TMNP modifies acid-insensitive T7 peptides and MG1 peptides.

### 2.4 *In vitro* release of resveratrol

Res@TcMNP/ASO in a dialysis bag (MWCO3500) was put in HEPES (pH = 7.4, 6.5, and 5.5 respectively) with 0.5% Tween-80 under horizontal shaking (100 rpm) at 37 °C. At predetermined times, resveratrol's concentration in the release medium was tested by HPLC at 306 nm (Agilent, Japan).

### 2.5 Cellular uptake and Endol/lysosomal escape assay

bEnd.3 and BV-2 cells were cultured with Cou-6-loaded NPs (each containing equal amounts of Cou-6) for 1 and 4 h for cellular uptake assay. bEnd.3 cells were cultured with Cou-6-loaded NPs for 4 h and stained with LysoTracker Red for endo/lysosomal escape assay.

### 2.6 *In vitro* BBB permeability and microglia targeting assay

bEnd.3 cells were cultured in Transwell plates' upper chambers (24-well; pore size: 3 μm; Corning, USA) to form a single-layer BBB model. FITC-labeled NPs (each containing equal amounts of FITC) were added directly to the upper chamber. Lower chambers’ fluorescence intensity was tested by a microplate reader to quantify BBB penetration efficiency.

Besides, BV-2 cells were cultured in the lower chamber to form a two-layer BBB model. bEnd.3 cells were incubated with FITC-labeled NPs (each containing equal amounts of FITC) for 6 h. Afterward, the BV-2 cells' nucleus in the lower chamber was stained with DAPI. NPs’ ability to permeate BBB and internalized by BV-2 cells was observed under the confocal laser scanning microscope (CLSM, Leica, DMi8, Germany).

### 2.7 *In vivo* imaging

Mice were injected with the DiD-loaded NPs (each containing equal amounts of DiD). Images were taken at 2, 4, 6, 8, 12, and 24 h after injection with Lumina III Imaging System (PerkinElmer, USA). Brains and other main organs were separated to take *ex vivo* fluorescence images. Use IVIS for semiquantitative analysis of the region of interest (ROI) analysis. Brain sections were labeled with the Iba1 antibody.

### 2.8 Anti-oxidative stress experiment *in vitro*

BV-2 cells were pre-incubated with 100 × 10^−6^ mol/L TBH for 6 h, incubated with resveratrol-loaded NPs (each containing equal amounts of resveratrol) for 24 h, and stained with 2′,7′-dichlorodihydrofluorescein diacetate (DCFH-DA, 10 μL, 10 μmol/L) at 37 °C for 20 min.

### 2.9 *In vitro* phagocytosis assay

BV-2 cells were pre-treated with ASO-loaded NPs (each containing equal amounts of ASO) for 24 h and incubated with Fluoresbrite YG Microspheres (Polysciences) (1:1000) for 2 h.

### 2.10 Investigation of microglia polarization *in vitro*

BV-2 cells were pre-incubated with LPS (1 μg/mL) to induce a pro-inflammatory state and further incubated with drug-loaded NPs for 24 h. The cells were fixed, permeabilized, digested to the cell suspension, and labeled with CD206 and CD86 antibodies, respectively. Meanwhile, the culture medium was saved to quantify cytokines with ELISA kits.

### 2.11 Neuroprotective assessment *in vitro*

The above media were collected as a conditioned medium and were incubated with SH-SY5Y cells. Cell viability and ROS level were measured. The cytoskeleton of SH-SY5Y cells was visualized by immunostaining using *β*-tubulin antibody.

### 2.12 Treatment of APP/PS1 mice

Ten-month-old APP/PS1 mice were randomly assigned to A, B, C, and D (10 mice per group), respectively representing Res@TcMNP/ASO, ResMNP/NC, TcMNP/ASO, and AD. Wild-type C57BL/6 mice were set as a control. Mice were injected intravenously with drug-loaded NPs (2 mg/kg resveratrol) every five days for a total of seven times.

### 2.13 Mechanism explorations *in vivo*

After the behavioral experiment, serum samples were collected from the mice for biochemical analysis and ELISA. Additionally, mice's brains and main organs were harvested for H&E or Nissl staining. Immunofluorescence staining of A*β*, Iba1, GFAP, and NeuN was performed on brain slices of different groups. Immunohistochemistry was conducted to observe the A*β* plaques and p-tau. Proteins in the brain were extracted for Western blot and ELISA.

### 2.14 Statistical analysis

Statistical analysis was processed by GraphPad Prism (version 8, CA). Data are expressed as mean ± standard deviation (SD). The unpaired Student's *t*-test was employed for comparing two groups, and the one-way ANOVA with Tukey's or Dunnett's test was employed for comparing multiple groups. A significance level of *P* < 0.05 indicated a statistically significant difference, and ∗*P* < 0.05, ∗∗*P* < 0.01, ∗∗∗*P* < 0.001.

## 3 Results and discussion

### 3.1 Synthesis and characterization of nanomodulators

**Figure 1:**
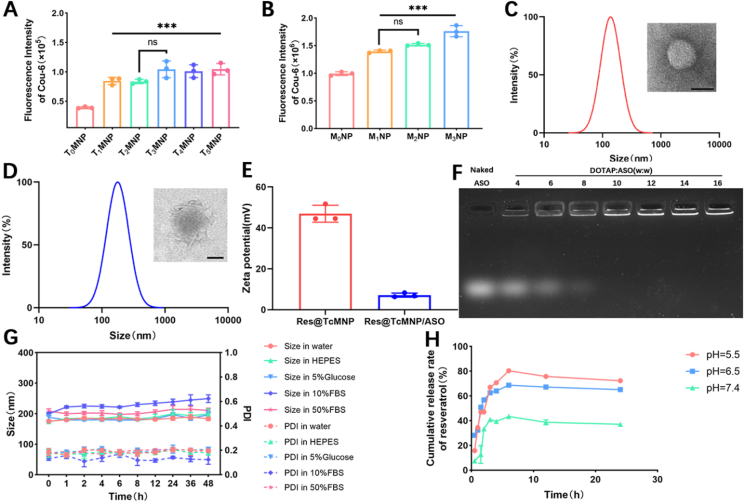
Synthesis and characterization of nanomodulators. (A) Uptake of bEnd.3 cells incubating with D-T7 modified NPs for 4 h T_0_MNP-T_5_MNP represent MNP containing 0%, 1%, 2%, 3%, 4%, or 5% DSPE-PEG3400-T7, respectively. (B) Uptake of BV-2 cells incubating with MG1-modified NPs for 4 h M_0_NP-M_3_NP represent NP containing 0%, 1%, 2%, or 3% DSPE-PEG3400-T7, respectively. DLS analysis and TEM image of (C) Res@TcMNP and (D) Res@TcMNP/ASO, scale bar = 100 nm. (E) Zeta potential of Res@TcMNP and Res@TcMNP/ASO. (F) Images of agarose gel electrophoresis of Res@TcMNP/ASO. (G) Stability of Res@TcMNP/ASO in water, HEPES (pH 7.4), 5% glucose, DMEM (10% FBS), and DMEM (50% FBS). (H) Resveratrol's cumulative release rate from Res@TcMNP/ASO in HEPES (pH = 7.4, 6.5, 5.5). Data are presented as mean ± SD (*n* = 3). ∗*P* < 0.05, ∗∗*P* < 0.01, ∗∗∗*P* < 0.001, and ns, not significant.

Incorporating no more than 5% (*mol/mol*) pegylated lipids can reduce nonspecific liposome–cell interactions^48^, ^49^, ^50^. To equip NPs with penetrating the BBB and targeting microglia ability, DSPE-PEG2000-MG1, DSPE-PEG3400-T7, and DSPE-hyd-PEG3400-T7 were synthesized and demonstrated by MALDI-TOF-MS (Supporting Information Figs. S1A–S1C, S2A–S2C, and S3A–S3C). The acid cleavability of DSPE-hyd-PEG3400-T7 and the acid stability of DSPE-PEG3400-T7 were also proved (Figs. S2D and S3D). To optimize D-T7 and MG1 peptides' modification density, we conducted bEnd.3 and BV-2 cellular uptake experiments (Fig. 1A and B). T_1_MNP was more efficient than T_0_MNP, while modification with more D-T7 showed no significant increase in uptake. The same trend occurred with the screening of MG1 peptides. Thus, TcMNP with 1% D-T7 and 1% MG1 modification was used in the following experiments. Then we prepared Res@TcMNP/ASO (Scheme 1A). The hydrodynamic size, polydispersity index (PDI), and zeta potential of Res@TcMNP were 123.42 ± 11.82 nm, 0.26 ± 0.08, and 46.94 ± 4.16 mV with spherical structures (Fig. 1C and E). Compared with TcMNP, Res@TcMNP showed a strong resveratrol characteristic absorption peak at 306 nm, indicating that resveratrol was successfully encapsulated in TcMNP (Supporting Information Fig. S4). The encapsulation efficiency and loading capacity of resveratrol were 88.88 ± 3.34% and 5.33 ± 0.19%, respectively. TREM2-lowing ASO was completely adsorbed by TcMNP with N/P of 10 through electrostatic interaction (Fig. 1F). The hydrodynamic size was about 175.33 ± 2.30 nm with a transparent layer on the surface of TcMNP, a little increased compared with that of Res@TcMNP (Fig. 1D). The zeta potential was partially neutralized, which also verified the efficiency incorporation of ASO (Fig. 1E). Besides, colloidal stability is a key indicator for NPs’ wide use^51^. Serum stability is also critical in gene delivery^52^^,^^53^. During the 48 h incubation, the hydrodynamic size and PDI of Res@TcMNP/ASO did not change significantly, indicating its high stability (Fig. 1G). *In vitro,* release profiles showed that under physiological conditions, resveratrol was slowly released, showing the ability of TcMNP to stably load resveratrol during circulation. In an acidic environment, the release rate of resveratrol increased markedly and up to 80% after 6 h (Fig. 1H). Notably, the AD brain presents a mildly acidic microenvironment^54^. So, Res@TcMNP/ASO could selectively release resveratrol in the pathological environment of AD, which is beneficial for AD-targeting delivery.

### 3.2 *In vitro* cellular level evaluation

**Figure 2:**
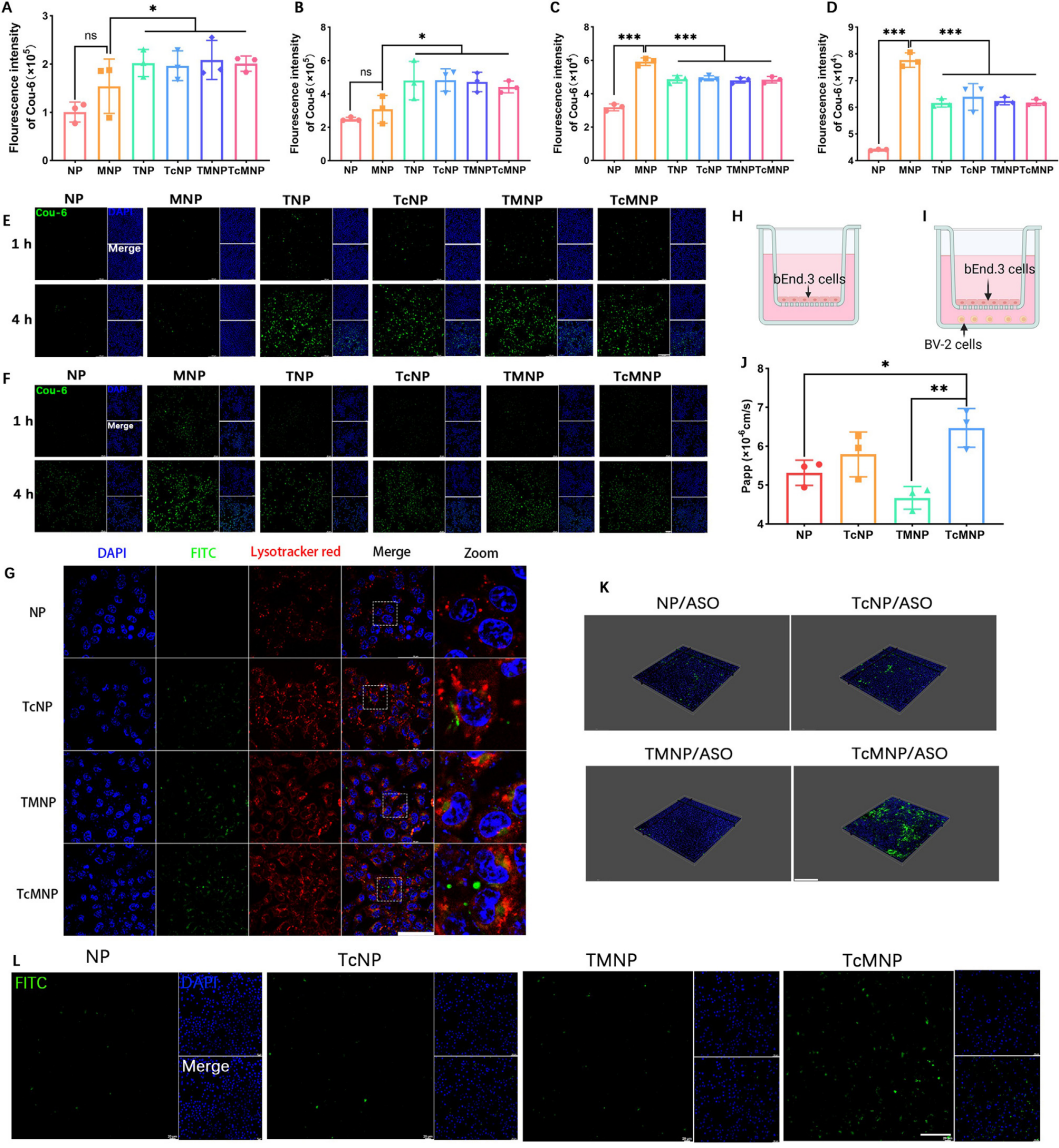
Intracellular behavior studies. Quantitative uptake of different NPs by bEnd.3 cells at 1 h (A) and 4 h (B). Quantitative uptake of different NPs by BV-2 cells at 1 h (C) and 4 h (D). CLSM imaging of NPs uptaken by bEnd.3 cells (E) and BV-2 cells (F). Scale bar = 100 μm. (G) Co-localization of NPs and lysosomes in bEnd.3 cells. Green, different Cou-6-loaded NPs; Red, LysoTracker; Blue, DAPI. Scale bar = 50 μm. Diagram of *in vitro* single-layer (H) and two-layer (I) BBB model. (J) Apparent permeability coefficient in the single-layer BBB model after incubating NPs for 4 h. (K) 3D confocal images of bEnd.3 monolayers. (L) CLSM images of lower chamber BV-2 cell uptake in the two-layer Transwell model. Scale bar = 100 μm. Data are presented as mean ± SD (*n* = 3). ∗*P* < 0.05, ∗∗*P* < 0.01, ∗∗∗*P* < 0.001, and ns, not significant.

Different Cou-6-loaded NPs were prepared to investigate the bEnd.3 and BV-2 cellular uptake. NPs modified with D-T7 peptides exhibited higher fluorescence intensity in bEnd.3 cells compared with NPs without D-T7 modification at 1 and 4 h. MNP exhibited brighter green fluorescence in BV-2 cells in a time-dependent behavior in contrast with NP. The uptake of TMNP was less than MNP, which may be due to the shadowing effect of long-chain PEG-T7 to short-chain PEG-MG1 (Fig. 2A–F).

The ability to penetrate the BBB is key to improving the treatment effect of AD drugs^55^. After being endocytosed into bEnd.3 cells, NPs need to escape from endo/lysosomes to facilitate penetrating BBB into the brain parenchyma, which was evaluated by co-localization of NPs (green) and lysosomes (red) in bEnd.3 cells (Fig. 2G). TMNP was prepared using DSPE-PEG3400-T7 instead of DSPE-hyd-PEG3400-T7 as a control. TMNP was mainly colocalized with endosomes/lysosomes. In contrast, most TcNP or TcMNP separated with endosomes/lysosomes due to the cleavage of the hydrazone bond. Then, a single-layer BBB model was used to assess the transmembrane efficiency of TcMNP (Fig. 2H). TcMNP achieved the highest penetration efficacy attributed to surface acid-sensitive D-T7. The fluorescent signal of the TMNP group in the lower chamber was weak, which may be due to its trapping in the lysosomes (Fig. 2J and K). We also established a two-layer BBB model to assess NP's microglial targeting ability (Fig. 2I). As expected, TcMNP exhibited enhanced microglial targeting after crossing the BBB (Fig. 2L). These findings underscore the enhanced efficacy of TcMNP for crossing BBB and targeting microglia.

### 3.3 *In vivo* BBB penetration and microglial targeting of TcMNP

**Figure 3:**
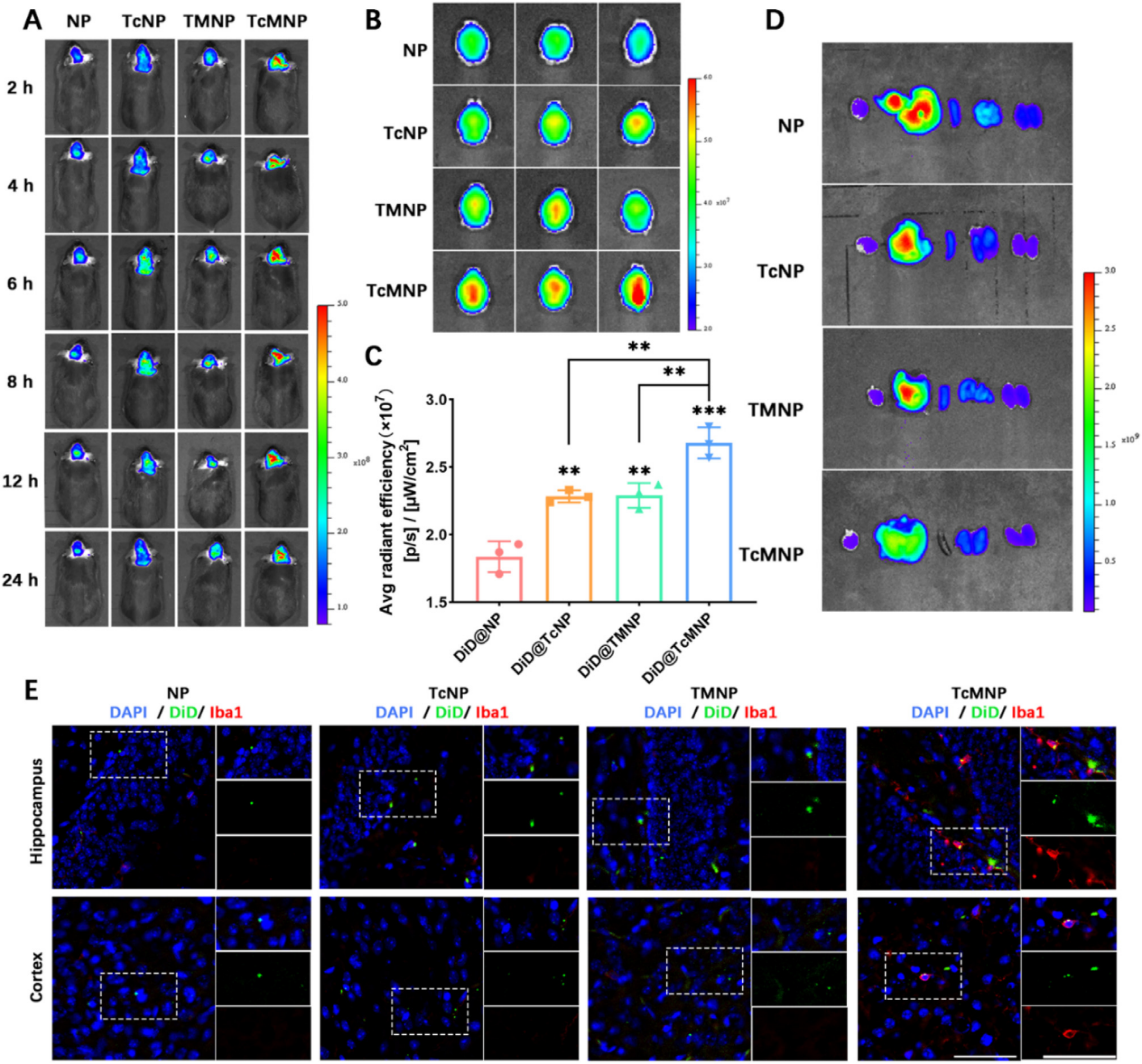
*In vivo* BBB penetration and microglial targeting of TcMNP. (A) Representative *in vivo* imaging of DiD-loaded NPs in mice. (B) *Ex vivo* images and (C) quantitative assessment of brains at 24 h. (D) Representative *ex vivo* imaging of major organs excised at 24 h. (E) Co-localization of microglia and NPs in brain sections' CLSM images. Green, DiD; Blue, DAPI; Red, microglia. Scale bar = 50 μm. Data are presented as mean ± SD (*n* = 3). ∗*P* < 0.05, ∗∗*P* < 0.01, ∗∗∗*P* < 0.001, and ns, not significant.

Inspired by the above *in vitro* experiment, mice injected with DiD-loaded NPs were monitored by a living fluorescence imaging system (Fig. 3A, Supporting Information Fig. S5). *In vivo*, fluorescence images showed that NPs modified with D-T7 had greater brain storage capacity than those without D-T7. TcMNP was more stored in the brain than TMNP, which may be attributed to the acid-cleavable D-T7. In addition, the fluorescence signal of the TcNP group was much lower than that of the TcMNP group, indicating the superiority of the cascade targeting strategy. *Ex vivo* images of major organs at 24 h showed that TcMNP had the strongest brain accumulation capacity. However, the distribution of other NPs was predominantly observed in the liver, due to its role as the primary metabolic organ (Fig. 3B–D and Supporting Information Fig. S6). The results of brain slices were also consistent with the above. Moreover, the microglia were further stained by the Iba1 (microglia marker) antibody. Most TcMNPs selectively targeted microglia (Fig. 3E). These results showed that DiD@TcMNP/ASO efficiently penetrated BBB and targeted microglia in APP/PS1 mice, laying the foundation for subsequent treatment.

### 3.4 *In vitro* synergistic inhibition of chronically activated microglia-mediated neuroinflammation

**Figure 4:**
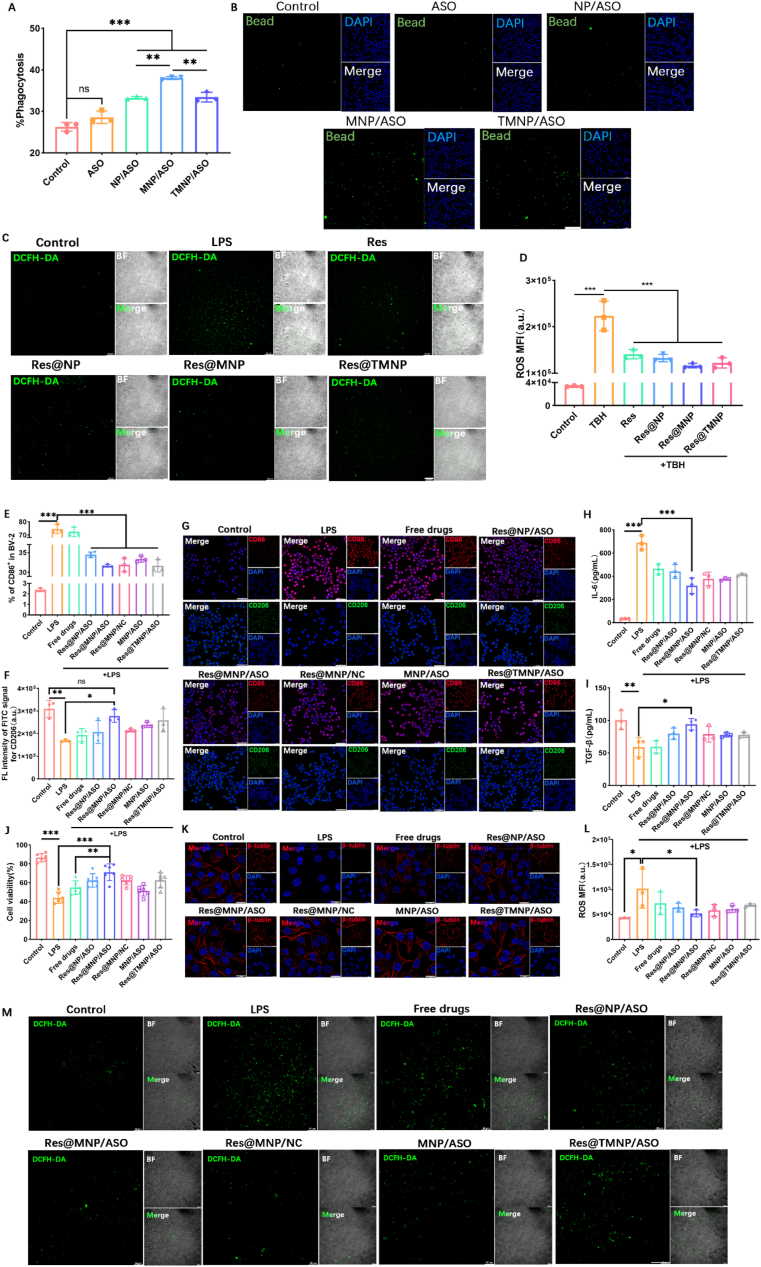
*In vitro* synergistic inhibition of chronically activated microglia-mediated neuroinflammation. (A) Quantitative (*n* = 3) and (B) qualitative analysis of phagocytosis of different NPs-treated BV-2 cells. Intracellular ROS of different NPs-treated BV-2 cells was observed *via* (C) CLSM and detected by (D) flow cytometry (*n* = 3). (E) CD86 and (F) CD206 expression of different NPs-treated BV-2 cells measured *via* flow cytometry (*n* = 3) and (G) CLSM. Scale bar = 50 μm. NC referred to the scrambled ASO which was used as a negative control. Levels of (H) IL-6 and (I) TGF-*β* in the supernatants of BV-2 cells (*n* = 3). (J) SH-SY5Y cell viabilities (*n* = 6) and (K) CLSM images of the cytoskeleton of SH-SY5Y cells treated with the supernatants of different NPs-treated BV-2 cells. Scale bar = 20 μm. Intracellular ROS of SH-SY5Y cells treated with the supernatants of different NPs-treated BV-2 cells *via* (L) flow cytometry (*n* = 3) and (M) CLSM. Scale bar = 100 μm. Data are presented as mean ± SD. ∗*P* < 0.05, ∗∗*P* < 0.01, ∗∗∗*P* < 0.001, and ns, not significant.

First, we explored whether TREM2-lowing ASO is beneficial in restoring the immune function of microglia by down-regulating TREM2. The expression of TREM2 in MNP/ASO-treated BV-2 cells was significantly decreased (Supporting Information Fig. S7). Then, different NPs-treated BV-2 cells were incubated with fluorescently labeled beads for 2 h. MNP/ASO-treated BV-2 cells exhibited maximal green signals, showing the restored phagocytic activity of microglial following ASO-mediated TREM2 reduction (Fig. 4A and B).

To assess the antioxidant capacity of NPs, BV-2 cells were pre-incubated with TBH. ROS signal in the Res@MNP group was significantly attenuated and approached the control. Res@MNP showed better ROS scavenging efficacy and low toxicity than the free resveratrol group because of its target ability to promote the internalization of resveratrol (Fig. 4C and D).

BV-2 cells were pre-incubated with LPS for 24 h, a common M1 phenotype inducer^56^. Co-incubation with LPS elevated the CD86 expression and the release of IL-6. The Res@MNP/ASO group had the highest polarization reversal efficiency with reduced IL-6 production and increased TGF-*β* levels, remarkably superior to other NPs in suppressing inflammation (Fig. 4E–I).

Inhibiting neuroinflammation in AD may alleviate misfolded protein-induced neurotoxicity^57^. An MTT assay was utilized to evaluate neuron viability with pre-incubating with the above supernatants of microglia (Fig. 4J). SH-SY5Y cells in the LPS group were damaged, and the cell viability decreased to about 40%. In contrast, the cell viability in the Res@MNP/ASO group was up to 70%. Microtubule stability and microfilament homeostasis in the neuronal cytoskeleton play an important role in axon regeneration^58^. The increase in fluorescence signal observed in *β*-tubulin staining further showed the restoration of the cytoskeletal structure in SH-SY5Y cells (Fig. 4K). Besides, the ROS level of SH-SY5Y cells in Res@TcMNP/ASO was significantly decreased (Fig. 4L and M).

These results showed that Res@MNP/ASO exhibited excellent efficacy in attenuating AD pathology *in vitro*. In detail, this nanomodulator could mediate the microglia-targeting delivery of TREM2-lowing ASO and resveratrol to restore the microglial phagocytic ability and eliminate the immune stimulation of ROS respectively, thus synergistically promoting the anti-inflammatory transformation of microglia and protecting neurons in the brain. This lays a solid foundation for *in vivo* drug efficacy and behavioral investigations.

### 3.5 Rescue the memory deficits by Res@TcMNP/ASO in APP/PS1 mice

**Figure 5:**
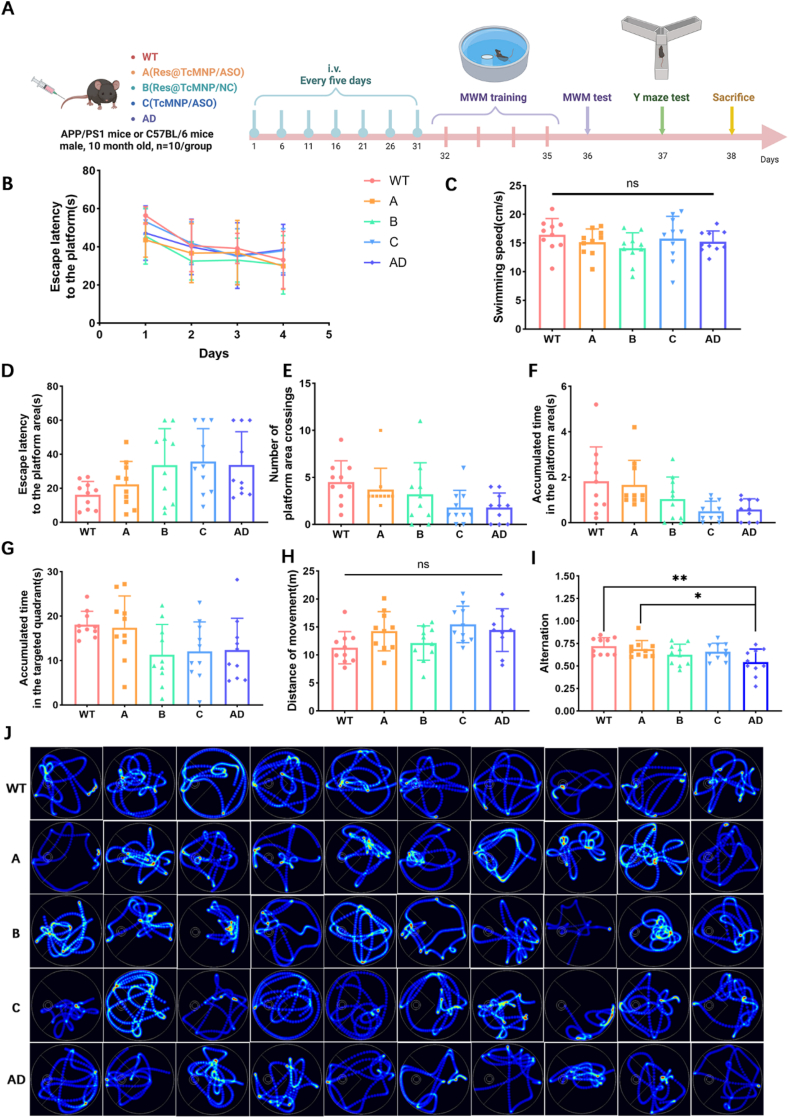
Behavioral studies. (A) Diagram of the experimental timeline. (B) The curve of escape latencies during training's first 4 days. (C) Swimming speeds in different groups. (D) The latency to reach the platform area. (E) Numbers of platform area crossings. (F) The accumulated time in the platform area. (G) The accumulated time in the targeted quadrant. (H) Distances of movement in different groups. (I) The spontaneous alternation rate for different groups. (J) Trajectory heat maps of different groups. Data are presented as mean ± SD (*n* = 10). ∗*P* < 0.05, ∗∗∗*P* < 0.01, ∗∗∗*P* < 0.001, and ns, not significant.

We further evaluate the immunotherapy effects of NPs on APP/PS1 mice (Fig. 5A). The treatment began at a late stage in the disease progression at the age of 10 months where immune hyperactivation is reported to already form in the brain in this model^59^. The Morris water maze (MWM) results showed the escape latency of the AD group decreased slightly on the first three days, and rebounded on the fourth day during the locomotor navigation phase from Day 1 to Day 4, suggesting severe memory deficits. Importantly, the escape latency was significantly reduced after Res@TcMNP/ASO treatment, showing a similar trend to WT mice (Fig. 5B). In the space exploration experiment, no statistical difference was found in the average swimming speed of the five groups, indicating no motor deficits (Fig. 5C). The swimming test track of the mice showed that after Res@TcMNP/ASO treatment, APP/PS1 mice preferentially moved toward the initial platform area, showing marked improvement in the searching strategy (Fig. 5J, Supporting Information Fig. S8). More importantly, Res@TcMNP/ASO-treated APP/PS1 mice showed the shortest escape latency, crossed the platform area most times, and spent the longest time in the target quadrant and platform area among the five groups, showing a strong desire to explore and the ability to learn (Fig. 5D–G). Furthermore, the Y-maze test results showed that no prominent differences were found in movement distance by these groups (Fig. 5H). The spontaneous alternation rate in APP/PS1 mice was decreased, while the spontaneous alternation rate in Res@TcMNP/ASO group was significantly improved and reached the level of WT mice (Fig. 5I). The above experimental results convincingly verified that Res@TcMNP/ASO exerted the most prominent effects for attenuating spatial memory deficits in AD mice and enhancing AD therapy efficiency.

### 3.6 The mechanism of synergistically microglial immune reprogramming modified by Res@TcMNP/ASO

**Figure 6:**
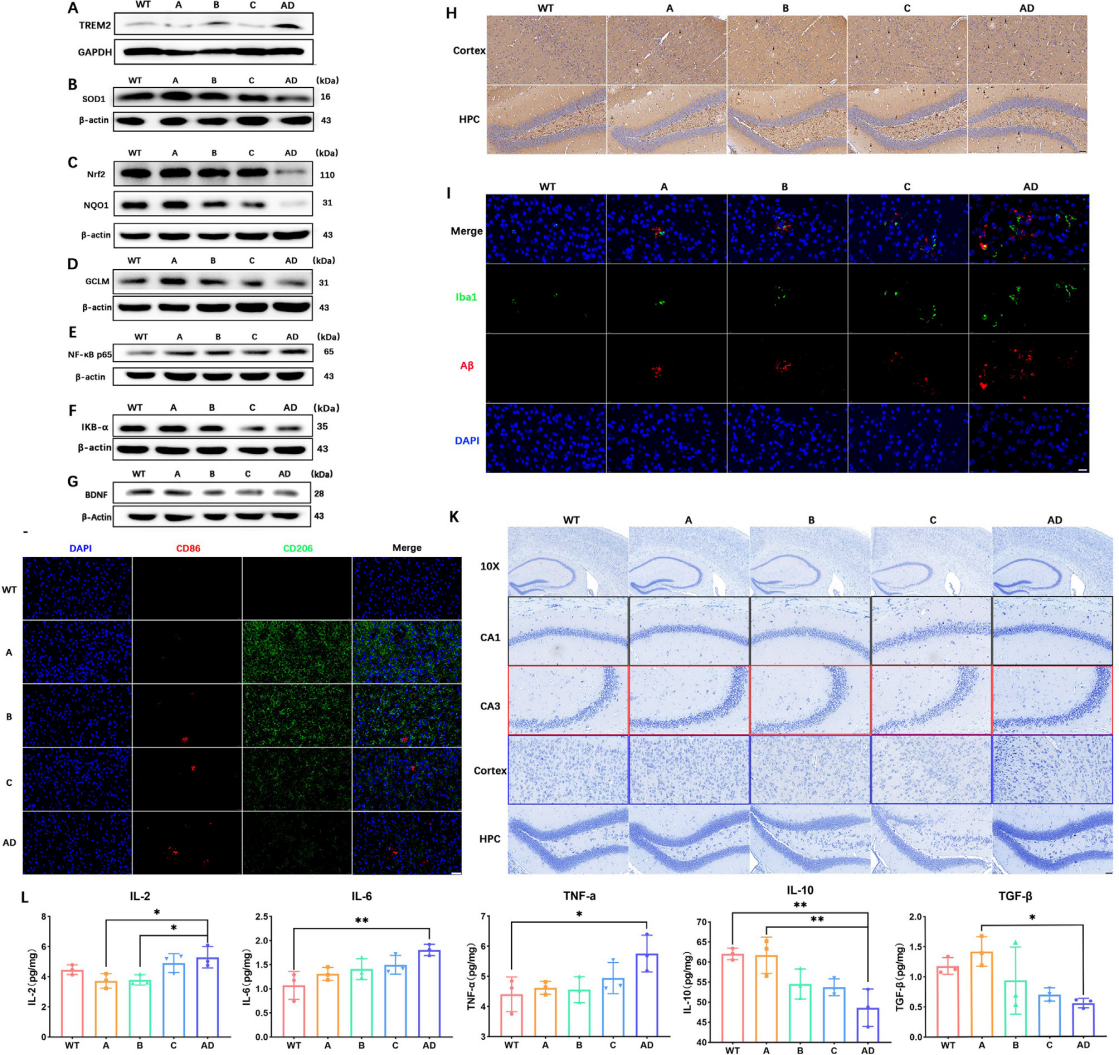
The mechanism of synergistically immune reprogramming of microglia modified by Res@TcMNP/ASO. Western blot assay detecting the (A) TREM2, (B) SOD1, (C) Nrf2, NQO1, (D) GCLM, (E) NF-*κ*B p65, (F) IKB-*α*, and (G) BDNF expression in the mice brain along with different treatments. (H) Representative immunohistochemical images of A*β* plaques in the mice brain along with different treatments. The black arrow shows where A*β* plaques occur. Scale bar = 40 μm. (I) Immunofluorescence images of A*β* plaques (red: A*β* plaques; blue: DAPI) and microglial (green: Iba1) in the mice brain. Scale bar = 20 μm. (J) Immunostaining of CD86 (M1 marker, red), and CD206 (M2 marker, green) in the cortex of APP/PS1 mice along with different treatments. Scale bar = 40 μm. (K) The representative Nissl staining of mice brain. Black boxes, CA1 area; Red boxes, CA3 area; Blue boxes, cortex area. First line, scale bar = 100 μm; other lines, scale bar = 40 μm. (L) Expression of IL-2, IL-6, TNF-*α*, IL-10, and TGF-*β* in the brain. Data are presented as mean ± SD (*n* = 3). ∗*P* < 0.05, ∗∗*P* < 0.01, ∗∗∗*P* < 0.001, and ns, not significant.

First, we investigated the downregulation of TREM2 protein *in vivo* (Fig. 6A**)**. The APP/PS1 mice exhibited an elevation in TREM2 levels, indicating the development of AD. The Res@TcMNP/ASO group exhibited a decrease in TREM2 levels compared to the Res@TcMNP/NC group. Next, we wondered whether Res@TcMNP/ASO could restore the phagocytic activity of microglia in APP/PS1 mice by downregulating TREM2. Immunohistochemistry and immunofluorescence results showed that Res@TcMNP/ASO effectively promoted A*β* plaques and p-tau deposits’ removal in the brain (Fig. 6H and I, Supporting Information Fig. S9).

The level of oxidative stress in the brain was evaluated, revealing that superoxide dismutase 1 (SOD1), an antioxidant protein, exhibited the highest levels in the Res@TcMNP/ASO group (Fig. 6B and Supporting Information Fig. S10). Moreover, the expression levels of nuclear factor erythroid-derived 2-like 2 (Nrf2) and its downstream antioxidant proteins, GCLM and NQO1, were markedly elevated (Fig. 6C and D and Supporting Information Figs. S11–S12). These results suggest that Res@TcMNP/ASO can activate the anti-oxidative stress pathway to protect cells from oxidative stress damage^60^.

Neuroinflammation due to the immune system's overactivation is closely related to the process of neurodegeneration^61^. Iba1(microglial activation marker) and GFAP (astrocytic activation marker) were detected by immunofluorescence to evaluate immune cells' abnormal activation in the brain. As depicted in Fig. 6I, microglia were activated in the brains of APP/PS1 mice. Res@TcMNP/ASO exhibited the most significant ability to reduce the overactivation of microglia while decreasing A*β* levels compared to all other NPs. Meanwhile, fewer GFAP-positive astrocytes were observed in the Res@TcMNP/ASO group than 10.13039/100020014AD group (Supporting Information Fig. S13). The images showing staining with CD86 (M1 microglia marker) and CD206 (M2 microglia marker) revealed that the microglia in the brain of WT mice were resting, while the Res@TcMNP/ASO treatment caused the microglial transition to an M2 phenotype (Fig. 6J). Similar results were observed in 10.13039/100007159Western blot analysis (Supporting Information Fig. S14). ELISA data showed a severe inflammatory response in the brain of APP/PS1 mice. Following the treatment, the Res@TcMNP/ASO group showed the lowest levels of inflammatory cytokines IL-2, IL-6, and TNF-*α* and the highest levels of anti-inflammatory cytokines IL-10 and TGF-*β* (Fig. 6L). IL-6 in peripheral blood was also reduced after treatment with Res@TcMNP/ASO (Supporting Information Fig. S15). To further understand the anti-inflammatory molecular mechanisms, we conducted additional research on the expression of NF-*κ*B p65 and its signaling pathway-related protein IKB-*α* (Fig. 6E and F and Supporting Information Figs. S16–S17). Res@TcMNP/ASO showed the lowest NF-*κ*B P65 and the highest IKB-*α*.

Finally, we verified the neuroprotective effects of Res@TcMNP/ASO. Immunofluorescence staining of hippocampal and cortical tissues showed that Res@TcMNP/ASO treatment showed the maximum fluorescence in neuron-specific nuclear proteins (NeuN, a neuronal marker) compared to the other groups (Supporting Information Fig. S18). The results of Nissl staining revealed pronounced neuronal damage in AD mice brains, characterized by dark purple staining indicative of cytoplasmic condensation, nucleus pyknosis, a significant decrease in cell density, and shrinkage of Nissl bodies. Additionally, fragmented neurons with irregular morphology were found in the cerebral cortex of the AD group. The neuronal damage was significantly alleviated by Res@TcMNP/ASO treatment. The neurons exhibited morphological integrity and orderly arrangement with a uniform light purple color. The Nissl bodies were well-organized (Fig. 6K). Similar results were obtained with hematoxylin and eosin (H&E) staining (Supporting Information Fig. S19). Brain-derived neurotrophic factor (BDNF) and postsynaptic density protein 95 (PSD95) levels were reduced in the 10.13039/100020014AD group and were upregulated after Res@TcMNP/ASO treatment (Fig. 6G and Supporting Information Figs. S20–S21).

These results validated that Res@TcMNP/ASO performed a remarkable therapy efficacy of attenuating AD pathology in the late APP/PS1 mice by synergistically inhibiting chronically activated microglia.

### 3.7 Safety evaluation of Res@TcMNP/ASO

Considering AD treatment's long-term characteristics, it is essential to ensure the high biocompatibility of administered NPs. Cell viability in bEnd.3 and BV-2 cells was more than 90% when the concentration of the Res@TcMNP/ASO was 100 μg/mL, showing negligible cytotoxicity at therapeutic concentrations *in vitro* (Supporting Information Figs. S22 and S23). The biocompatibility of Res@TcMNP/ASO was further examined *in vivo* by hemolysis rate, which showed that Res@TcMNP/ASO is highly blood-compatible (Supporting Information Fig. S24). Besides, the body weight of mice fluctuated within the normal range during the treatment period, indicating that the NPs have no obvious systemic toxicity (Supporting Information Fig. S25). Blood biochemical results showed that *in vivo* administration of Res@TcMNP/ASO had no significant effect on plasma alanine aminotransferase (ALT), plasma urea (BUN), creatinine (CREA), and aspartate aminotransferase (AST) levels, indicating no noticeable hepatorenal toxicity (Supporting Information Fig. S26). Additionally, the main organs of Res@TcMNP/ASO treated-APP/PS1 mice were normal without any pathological changes (Supporting Information Fig. S27).

## 4 Conclusions

In short, we designed a microglial-targeting nanomodulator to co-delivery resveratrol and TREM2-lowing ASO. By dual targeting peptides and acid-sensitive bonds, this nanomodulator can effectively escape from endo/lysosomes to cross the BBB and accumulate in M1-phenotype microglia in AD lesions. Res@TcMNP/ASO could modulate the chronic-activated microglia *via* two synergistic approaches: 1) resveratrol eliminates the immune stimulation of ROS and 2) TREM2-lowing ASO induces acute activation of microglia, restores microglial phagocytosis ability, thus synergistically suppressing neuroinflammation. The combined strategy prevented the crosstalk between chronically activated microglia and neuroinflammation in AD. Results showed that Res@TcMNP/ASO exhibits better treatment effects in late APP/PS1 mice than Res@TcMNP/NC and TcMNP/ASO. Our study suggests a promising strategy for flexible manipulation of microglial immune function to combat AD, providing a new horizon for AD immune therapy. More importantly, the specific microglia-targeting ability of TcMNP holds promise for manipulating microglial activity and preventing diseases associated with microglial dysfunction.

## Author contributions

Huile Gao and Juntao Fu designed the research. Ya Wei carried out the most of experiments and constructed pictures with assistance of Xue Xia, Xiaorong Wang, Wenqin Yang, Siqin He, Lulu Wang, and Yongke Chen. Ya Wei wrote the manuscript with assistance of Yang Zhou, Feng Chen, Hanmei Li, Fu Peng, Guobo Li and Zheng Xu. Huile Gao and Jintao Fu provided guidance on revising the manuscript. All the authors have read and approved the final manuscript.

## Conflicts of interest

The authors declare no conflict of interest.

## Appendix A Supplementary information

The following is the Supporting Information to this article.
