# Supplementary material for: Enhanced BBB penetration and microglia-targeting nanomodulator for the two-pronged modulation of chronically activated microglia-mediated neuroinflammation in Alzheimer's disease
Source: Acta Pharm Sin B. 2025 Jan 25;15(2):1098–111. doi: 10.1016/j.apsb.2025.01.015 (PMC11959930; doi:10.1016/j.apsb.2025.01.015)
Supplement: Multimedia component 1 [file mmc1.pdf]

Supporting Information for

Original article

Enhanced BBB penetration and microglia-targeting nanomodulator for the two-pronged modulation of chronically activated microglia-mediated neuroinflammation in Alzheimer's disease

Ya Wei[a, b], Xue Xia[b], Xiaorong Wang[b], Wenqin Yang[b], Siqin He[b], Lulu Wang[a], Yongke Chen[b], Yang Zhou[a], Feng Chen[c], Hanmei Li[d], Fu Peng[b], Guobo Li[b], Zheng Xu[e], Jintao Fu[a, *], Huile Gao[a,b, *]

[a]*Key Laboratory of Tropical Biological Resources of Ministry of Education, School of Pharmaceutical Sciences, Hainan University, Haikou 570200, China*

[b]*Key Laboratory of Drug-Targeting and Drug Delivery System of the Education Ministry, West China School of Pharmacy, Sichuan University, Chengdu 610041, China*

[c]*Department of Radiology, Hainan General Hospital (Hainan Affiliated Hospital of Hainan Medical University), Haikou, China*

[d]*School of Food and Biological Engineering, Chengdu University, Chengdu 610106, China*

[e]*State Key Laboratory of Biotherapy, West China Hospital, Sichuan University, Chengdu 610041, China*

Received 22 September 2024; received in revised form 5 December 2024; accepted 25 December 2024

*Corresponding authors.

E-mail addresses: gaohuile@scu.edu.cn (Huile Gao), fujintao@hainanu.edu.cn (Jintao Fu).

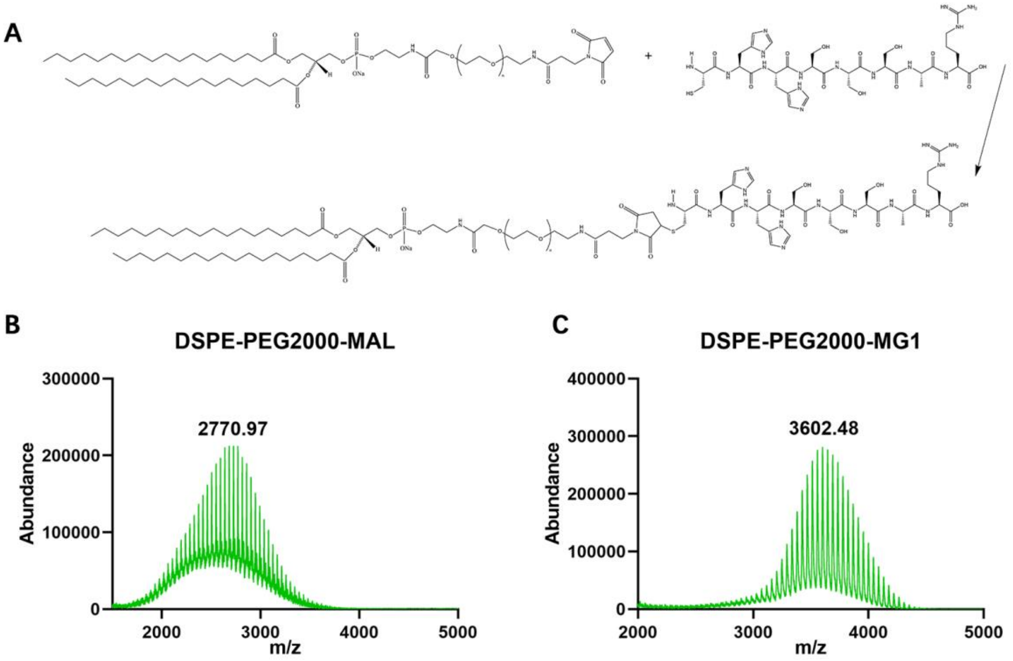

Figure S1 Characterization of DSPE-PEG2000-MG1. (A) Synthetic route of DSPE-PEG2000-MG1. MALDI-TOF-MS spectrum of DSPE-PEG2000-MAL (B) and DSPE-PEG2000-MG1 (C).

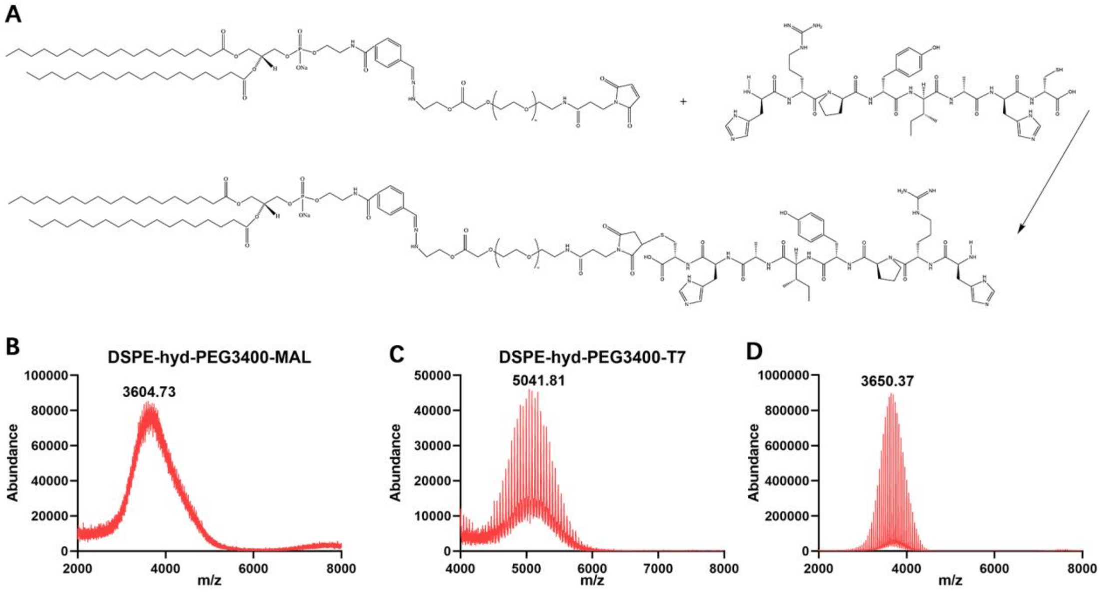

Figure S2 Characterization of DSPE-hyd-PEG3400-T7. (A) Synthetic route of DSPE-hyd-PEG3400-T7. MALDI-TOF-MS spectrum of DSPE-hyd-PEG3400-MAL (B) and DSPE-hyd-PEG3400-T7 (C) and DSPE-hyd-PEG3400-T7 incubated with pH = 5.5 buffer for 12 h (D).

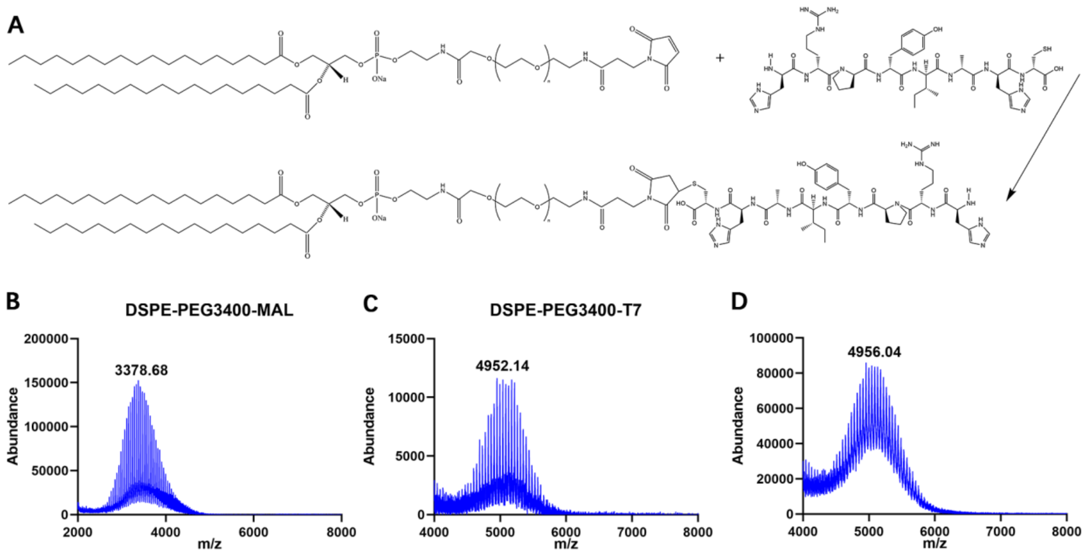

Figure S3 Characterization of DSPE-PEG3400-T7. (A) Synthetic route of DSPE-PEG3400-T7. MALDI-TOF-MS spectrum of DSPE-PEG3400-MAL (B), DSPE-PEG3400-T7 (C), and DSPE-PEG3400-T7 incubated with pH 5.5 buffer for 12 h (D).

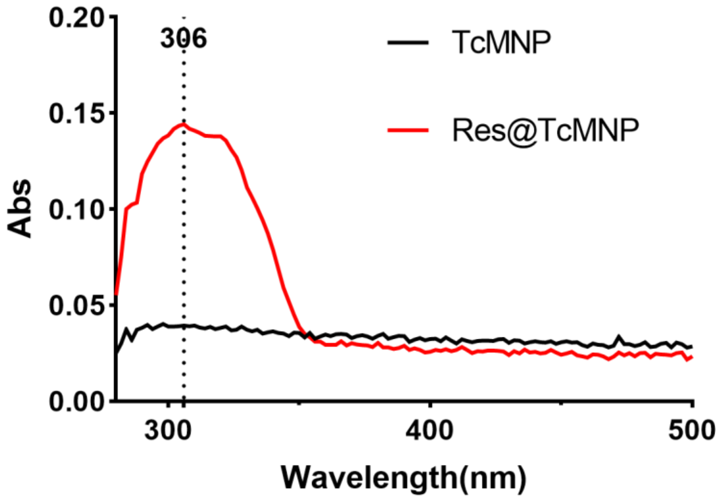

Figure S4 UV–Vis absorbance of TcMNP, and Res@TcMNP.

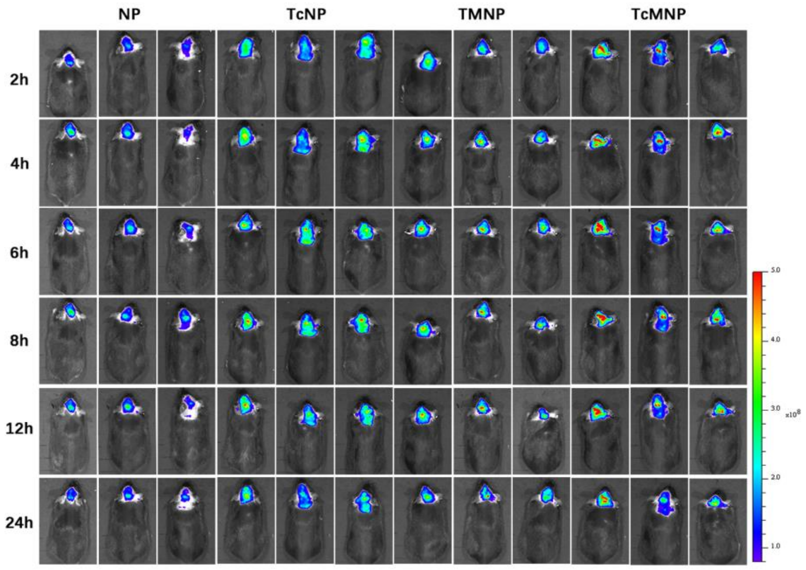

Figure S5 *In vivo* imaging of DiD-loaded NPs in the whole body of APP/PS1 AD mice at different time intervals.

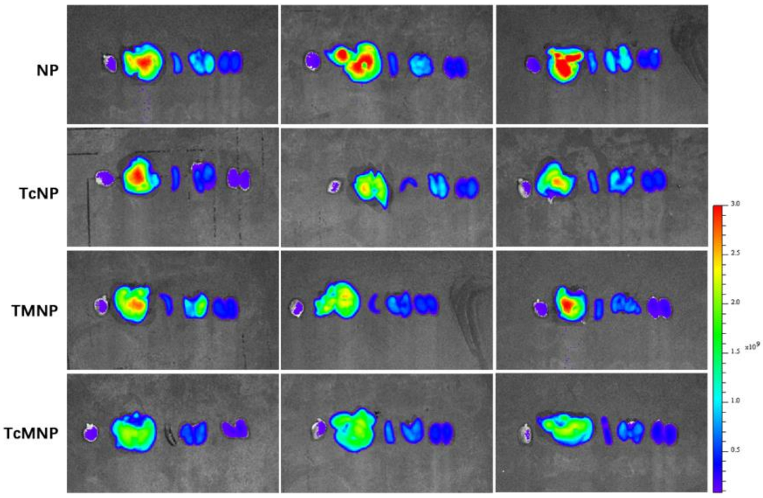

Figure S6 *Ex vivo* imaging of major organs (heart, liver, spleen, lung, kidney) excised at 24 h post administration.

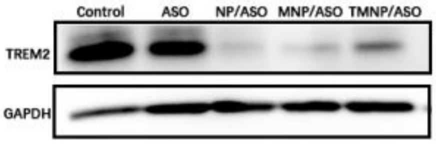

Figure S7 Western blot assay detecting the TREM2 expression in BV-2 cells along with different treatments.

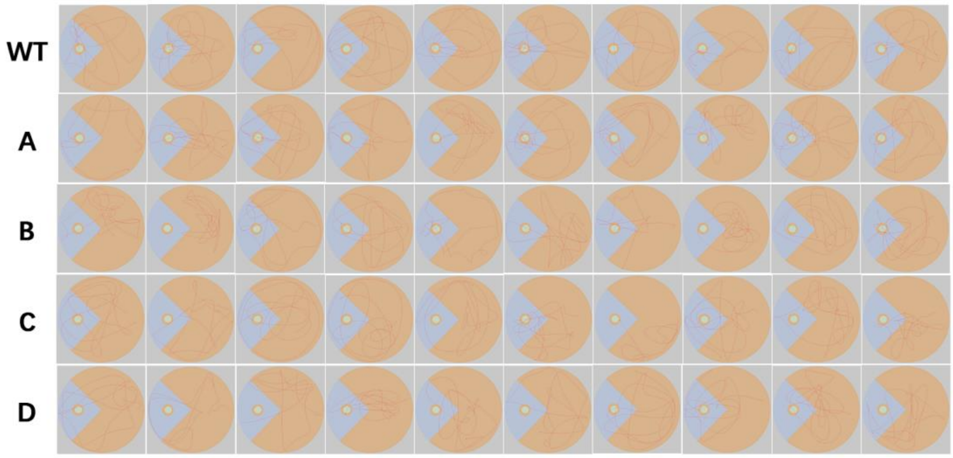

Figure S8 Representative swimming paths of different groups in the MWM test.

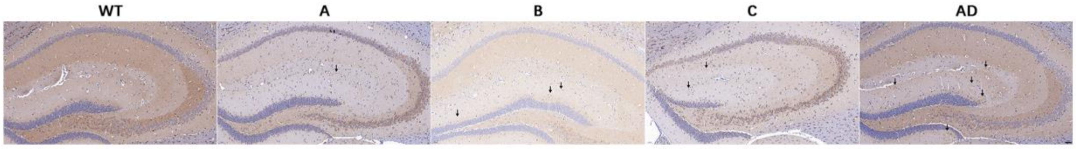

Figure S9 Representative images of p-tau stained by immunohistochemical in hippocampus of the mice brain along with different treatments. The black arrow shows where p-tau occurs. Scale bar =50 μm.

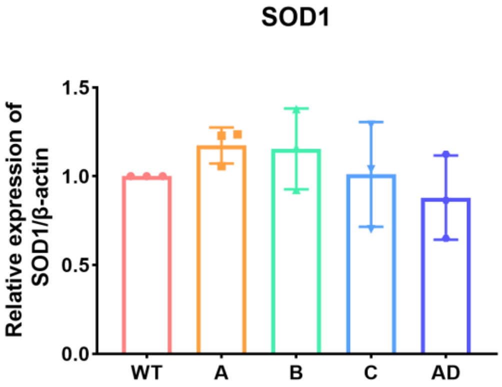

Figure S10 Quantification of SOD1 expression in the mice brain along with different treatments. Data were presented as mean ± SD (n = 3).

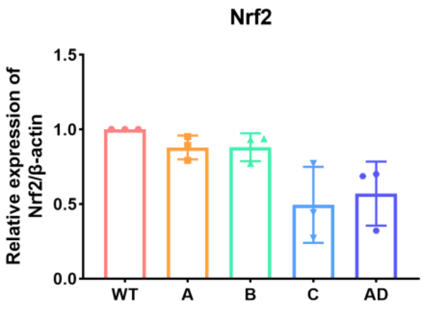

Figure S11 Quantification of Nrf2 expression in the mice brain along with different treatments. Data were presented as mean ± SD (n = 3).

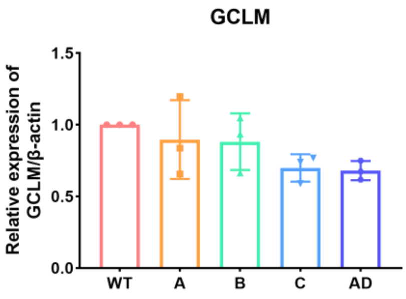

Figure S12 Quantification of GCLM expression in the mice brain along with different treatments. Data were presented as mean ± SD (n = 3).

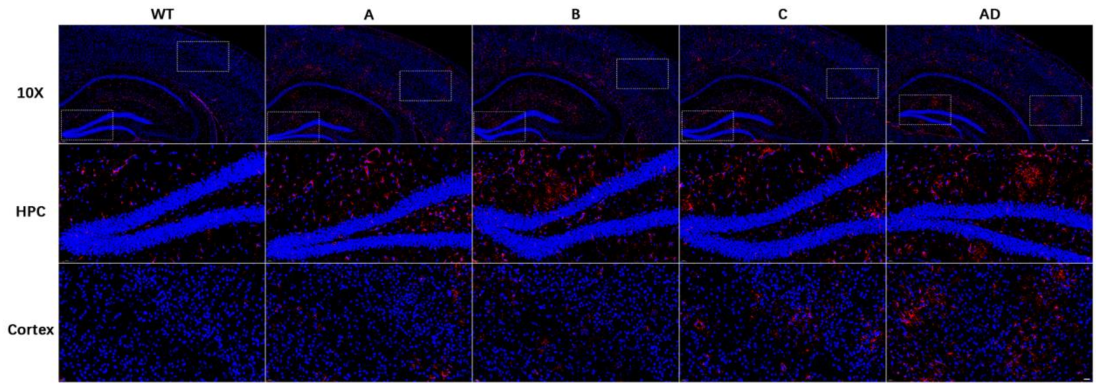

Figure S13 Immunofluorescence of GFAP in the hippocampus and cortex of the mice brain along with different treatments (Red: GFAP; blue: DAPI). Scale bar = 20 μm.

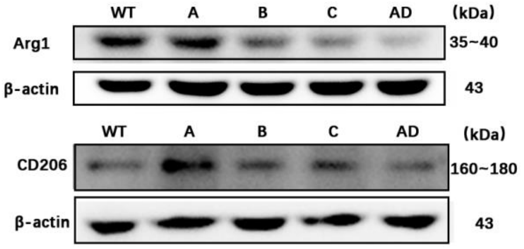

Figure S14 Western blot assay detecting the Arg1 and CD206 expression in the mice brain along with different treatments.

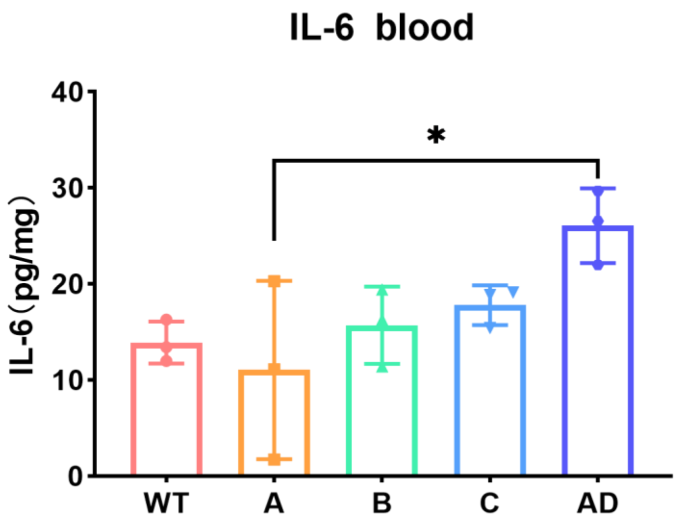

Figure S15 Expression of pro-inflammatory cytokines IL-6 in the blood along with different treatments.. Data were presented as mean ± SD (n = 3). P > 0.05, 0.01 ≤ P < 0.05, 0.001≤ P <0.01, and P < 0.001 were remarked with ns, *, **, and ***, respectively.

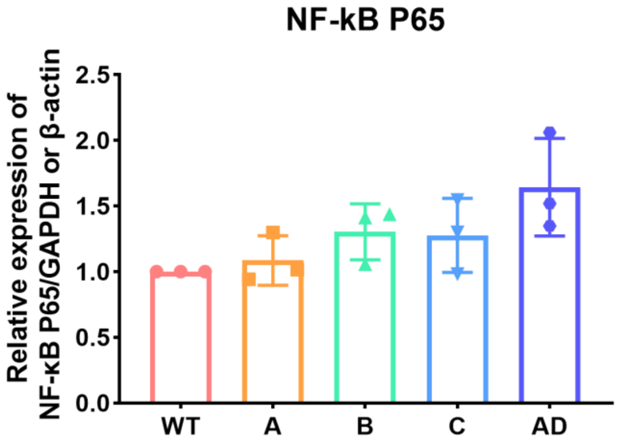

Figure S16 Quantification of NF-κB p65 expression in the mice brain along with different treatments. Data were presented as mean ± SD (n = 3).

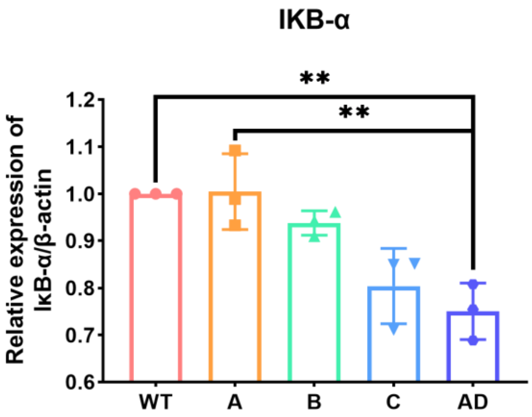

Figure S17 Quantification of IKB-*α* expression in the mice brain along with different treatments. Data were presented as mean ± SD (*n* = 3).

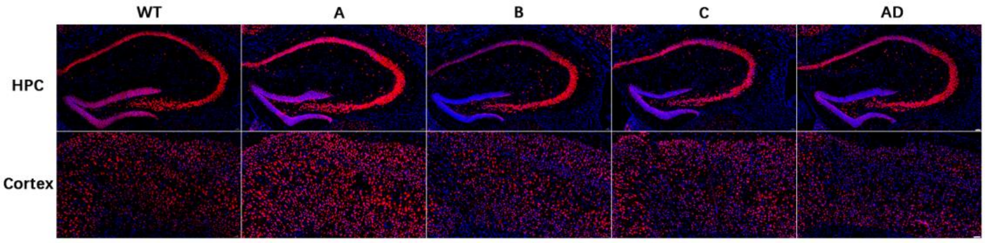

Figure S18 Immunofluorescence images of NeuN in the hippocampus and cortex of mice brain along with different treatments. Scale bar = 50 μm.

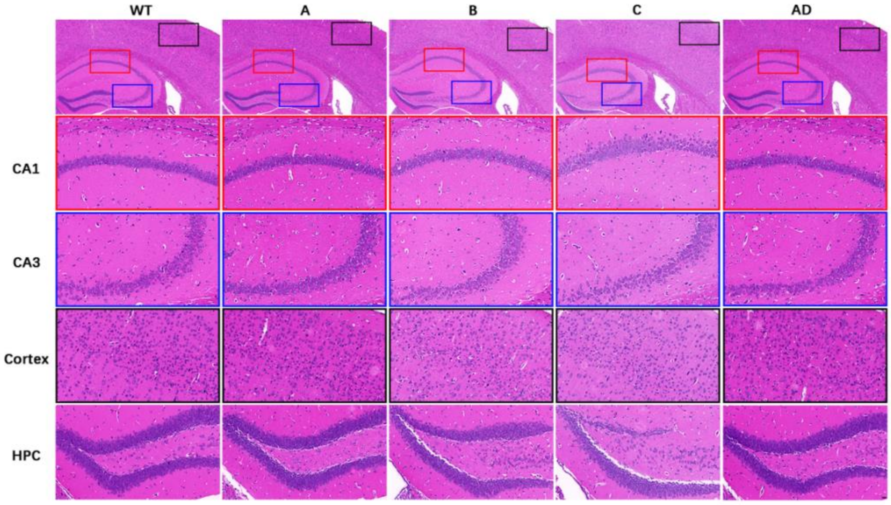

Figure S19 HE staining images of mice brain along with different treatments. The white boxes show the hippocampal CA1 area, the red boxes show the hippocampal CA1 area, the blue boxes show the hippocampal CAs area, the black boxes show the cortex area. First line: scale bar =

100 μm; other lines: scale bar = 20 μm.

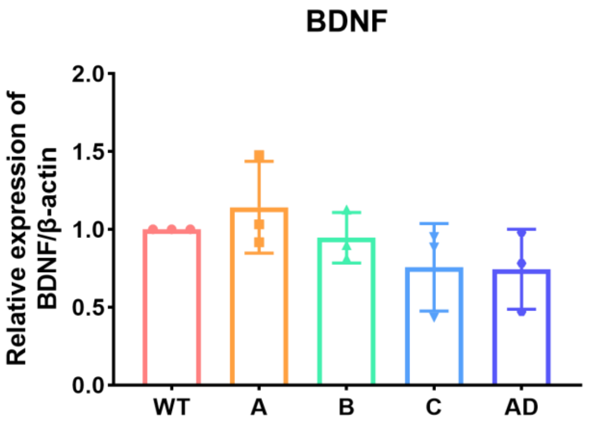

Figure S20 Quantification of BDNF expression in the mice brain along with different treatments. Data were presented as mean ± SD (n = 3).

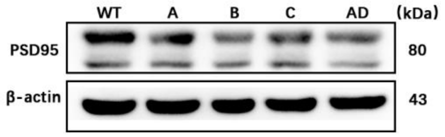

Figure S21 Western blot assay detecting the PSD95 expression in the mice brain along with different treatments.

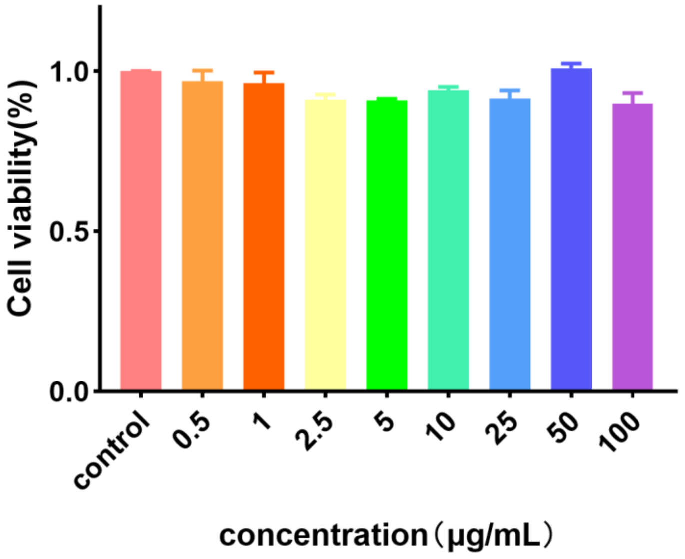

Figure S22 The bEnd.3 cell viability of Res@TcMNP/ASO measured by MTT assay. Data are presented as mean ± SD (n = 3).

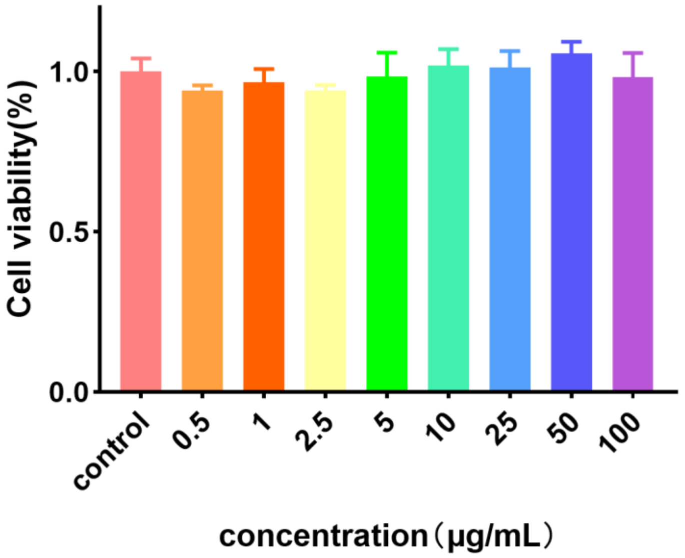

Figure S23 The BV-2 cell viability of Res@TcMNP/ASO measured by MTT assay. Data are presented as mean ± SD (n = 3).

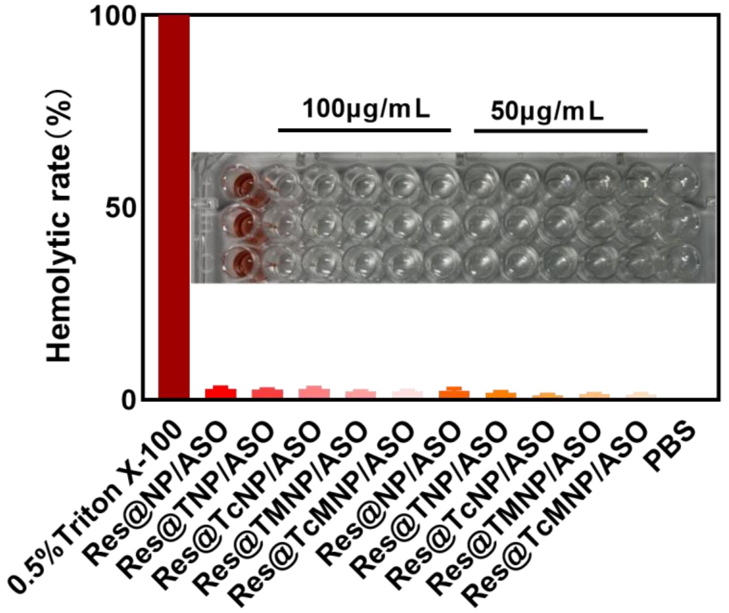

Figure S24 Hemolysis test of different NPs in different concentrations. Data are presented as mean ± SD (n = 3).

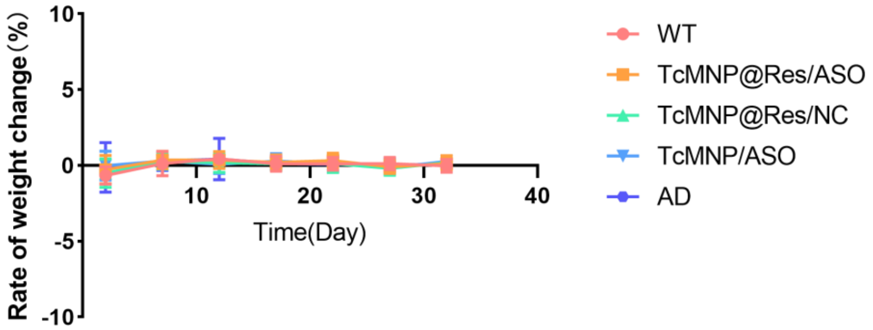

Figure S25 Rate of Body weight changes of WT and APP/PS1 mice during treatment. Data are presented as mean ± SD, n = 10.

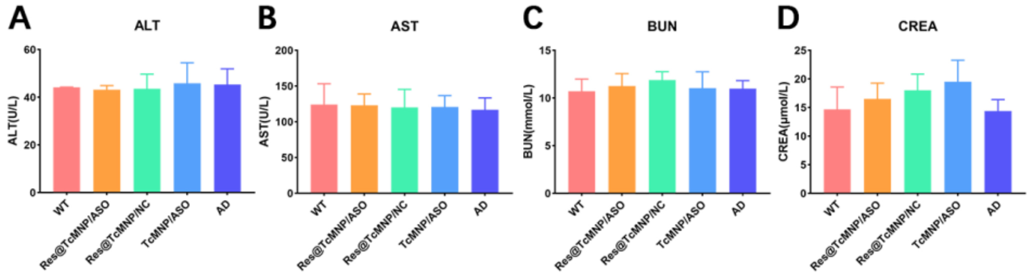

Figure S26 Blood chemistry examinations after treatment, including plasma alanine aminotransferase (ALT), aspartate aminotransferase (AST), plasma urea (BUN), and creatinine (CREA). Data are presented as mean ± SD, n = 4.

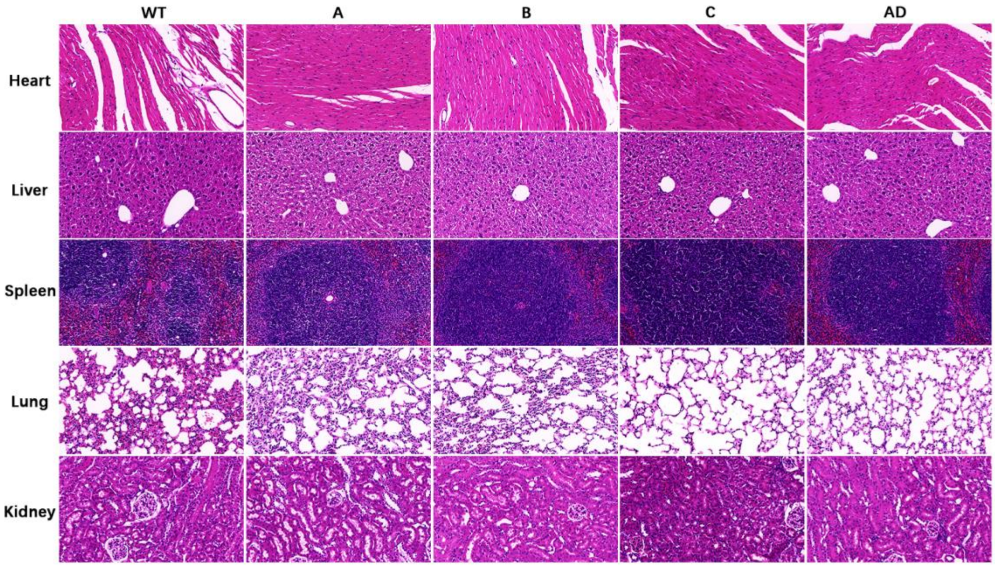

Figure S27 Representative images for HE staining in major organs from the mice along with different treatments. Scale bar = 20 μm.
